# Extraction of Chitin From Shrimp Shell by Successive Two-Step Fermentation of *Exiguobacterium profundum* and *Lactobacillus acidophilus*

**DOI:** 10.3389/fmicb.2021.677126

**Published:** 2021-09-14

**Authors:** Jingwen Xie, Wancui Xie, Jing Yu, Rongyu Xin, Zhenping Shi, Lin Song, Xihong Yang

**Affiliations:** ^1^College of Marine Science and Biological Engineering, Qingdao University of Science and Technology, Qingdao, China; ^2^Shandong Provincial Key Laboratory of Biochemical Engineering, Qingdao, China; ^3^Qingdao Keda Future Biotechnology Co., Ltd, Qingdao, China

**Keywords:** successive two-step fermentation, *Lactobacillus acidophilus*, *Exiguobacterium profundum*, chitin, demineralization, deproteinization, shrimp shell

## Abstract

As an environmentally friendly and efficient method, successive two-step fermentation has been applied for extracting chitin from shrimp shells. To screen out the microorganisms for fermentation, a protease-producing strain, *Exiguobacterium profundum*, and a lactic acid-producing strain, *Lactobacillus acidophilus*, were isolated from the traditional fermented shrimp paste. Chitin was extracted by successive two-step fermentation with these two strains, and 85.9 ± 1.2% of protein and 95 ± 3% of minerals were removed. The recovery and yield of chitin were 47.82 and 16.32%, respectively. Fourier transform infrared spectroscopy, X-ray diffraction, and scanning electron microscopy (SEM) were used to characterize the chitin. The crystallinity index was 54.37%, and the degree of deacetylation was 3.67%, which was lower than that of chitin extracted by the chemical method. These results indicated that successive two-step fermentation using these two bacterial strains could be applied to extract chitin. This work provides a suitable strategy for developing an effective method to extract chitin by microbial fermentation.

## Introduction

**FIGURE 1:**
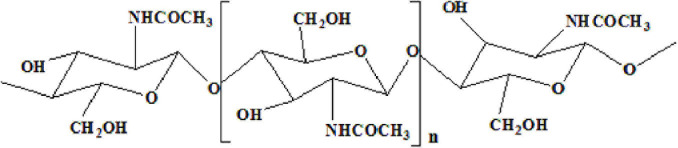
Fully acetylated chitin polymer chain.

Chitin, an insoluble linear homopolymer of β-(1→4)-linked-*N*-acetyl-D-glucosamine (Figure 1; Duan et al., 2012; Sharp, 2013), is the second-largest carbohydrate polymer in nature (Younes et al., 2014). It exhibits good biocompatibility (Sedaghat et al., 2017), antibacterial activity, and biodegradability (Mao et al., 2013), which enables its wide application in medicine, biotechnology, nutrition, and food processing (Oh et al., 2007). In recent decades, chitin from shrimp shells attracted extensive attention. Chemical treatment and enzymatic reactions have been extensively studied to prepare chitin (Xin et al., 2020). The process of extracting and preparing chitin from shrimp shells involves demineralization (DM), deproteinization (DP), deacetylation, and depolymerization (Fu et al., 2019). However, the use of harmful chemicals can affect the environment (Kim and Park, 2015). Due to the increasing use of commercial enzymes, high costs make it difficult to produce them on a large scale on an industrial scale (Bougatef, 2013). Therefore,

an effective and environmentally friendly technology can easily recover chitin without affecting the environment (Duan et al., 2012).

As an eco-friendly, technically flexible, and economically viable method, microbial fermentation has become a promising approach for extracting chitin. Protease from microorganisms has been successfully adopted for DP and DM during the process of chitin-rich marine crustacean shells. Previous studies reported about microorganisms, including *Lactobacillus plantarum* (Rao and Stevens, 2005), *Lactobacillus helveticus* (Arbia et al., 2013), *Pediococcus acidolactici* (Bhaskar et al., 2007), *Bacillus subtilis* (Sun and Mao, 2016), *Bacillus cereus* (Manni et al., 2010), *Bacillus licheniformis* (Hajji et al., 2015), *Pseudomonas aeruginosa* (Oh et al., 2007), and *Streptococcus thermophilus* (Mao et al., 2013). For example, *Lactobacillus* sp. B2 could remove 56% of protein and 88% of minerals from the crab wastes (Flores-Albino et al., 2012). *Bacillus* spp. could remove more than 80% of protein and less than 67% of mineral from the shrimp waste (Ghorbel-Bellaaj et al., 2012). *L. plantarum* 541 was used by Rao and Stevens (2005) to remove 66% of protein and 63% of minerals. However, pure chitin cannot be obtained by single-strain fermentation. Therefore, it is necessary to improve the removal efficiency of proteins and minerals in shrimp shell fermentation.

In order to improve the removal efficiency of proteins and minerals in chitin from shrimp shells, a successive two-step fermentation was employed in this study. A strain with high protease activity, *Exiguobacterium profundum*, and a stain capable of producing lactic acid, *L. acidophilus*, were isolated from a traditional fermented shrimp paste. The DP and DM efficiency were significantly improved, which indicates the applicable potential in chitin extraction.

## Materials and Methods

### Materials

Shrimp shells were purchased from Kaiping Road farmer’s market, Qingdao, China. The obtained shells were washed with running water for removing the shrimp residue and other impurities. Then the shells were washed with distilled water and dried. Finally, the dried samples were disintegrated with a grinder (Yongkang Platinum Metal Products Co., Ltd., China), and broken carefully into 2–3-mm sizes. *E. profundum* and *L. acidophilus* were screened out from fermented shrimp paste in previous experiments.

### Isolation and Identification of Microorganisms

Sample treatment and bacterial isolation were conducted according to Sun and Mao (2016) with minor modifications as follows: after diluting and dispersing, the solution was spread onto marine agar containing 2% (w/v) skim milk and MRS agar containing 2% (w/v) CaCO_3_ and incubated for 3 days. The colonies were identified as genera *Exiguobacterium* and *Lactobacillus* using traditional morphological methods. 16S rDNA sequence analysis was done to identify *E. profundum* and *L. acidophilus*. A phylogenetic tree was constructed using MEGA software (version 7.0) by the neighbor-joining method using the 16S rDNA sequences of selected and type strains (Sun and Mao, 2016).

### Culture Conditions

The bacterial strains were isolated from traditional fermented shrimp paste and stored in a 25% glycerol preservation tube at −80°C. For *E. profundum* fermentation, the culture medium used by Anbu et al. (2013) contained shrimp shells as the sole nitrogen source. For *L. acidophilus* fermentation, the medium (per 1000 ml distilled water) contained 10 g peptone, 5 g CH_3_COONa, 2 g ammonium citrate, 0.58 g MgSO_4_, 0.25 g MnSO_4_, 50 g glucose, 2.0 g K_2_HPO_4_, and 50 g/L shrimp shell (pH 6.2 ± 0.2). For successive two-step fermentation, the initial fermentation medium contained 10 g peptone, 5 g CH_3_COONa, 2 g ammonium citrate, 0.58 g MgSO_4_, 0.25 g MnSO_4_, 50 g glucose, 2.0 g K_2_HPO_4_, and 50 g/L shrimp shell (pH 6.2 ± 0.2), and the medium for replacement contained shrimp shells fermented by the first step containing 2.5 g glucose, 5.0 g NaCl, 2.5 g/L K_2_HPO_4_ (pH 7.3 ± 0.2).

### Fermentation

**FIGURE 2:**
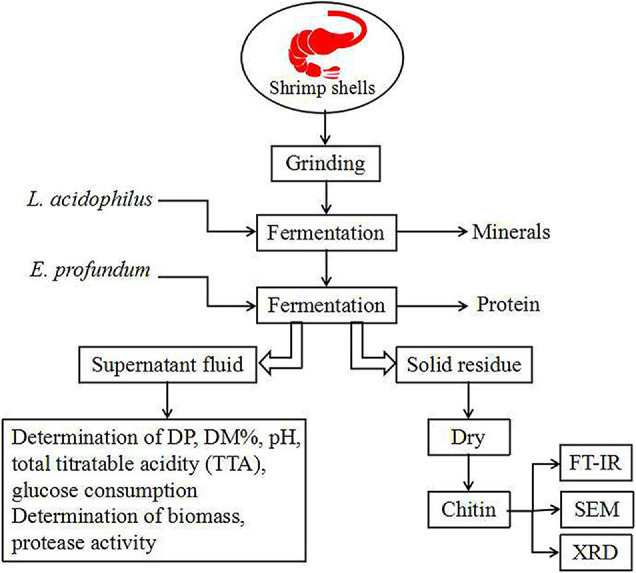
Flowsheet of the process for this study. DP, deproteination; DM, demineralization.

The experimental flowsheet was shown in Figure 2. For *L. acidophilus* fermentation, each 100-ml fermentation medium was seeded with *L. acidophilus* (10^8^–10^9^ CFU/ml) in a shaking incubator (140 rpm) at room temperature for 120 h with a fermented supernatant collected at 12-h intervals for growth curve statistics. The pH was determined using a pH meter (Leici, PHS-2F, Shanghai, China), and the total titratable acidity (TTA) of the supernatant was diluted with 0.1 mol/L NaOH (pH = 8.4) (Prameela et al., 2010). The glucose consumption was determined by the dinitrosalicylic acid (DNS) method. The biomass was determined by the plate counting method; in brief, biofilm solutions were serially diluted prior to plating, and three different dilutions were plated out in order to obtain at least one plate containing 20–200 CFUs. The total number of original colonies was obtained according to the multiple of dilution.

For *E. profundum* fermentation, fermentation conditions were similar to *L. acidophilus* but the fermented supernatant was collected at 24-h intervals. The protease activity was measured according to the method described by Kembhavi (1993). The soluble peptide in the supernatant fraction was measured using tyrosine as a reference (Todd, 1949). One unit of protease activity is defined as the amount of enzyme required to liberate 1 μmol of tyrosine per minute (Zhang et al., 2012).

For successive two-step fermentation, fermentation was carried out using *L. acidophilus* in the same manner. After 96 h, the fermentation medium was replaced (see section “Culture Conditions”), and *E. profundum* was used for fermentation. The fermented sample was filtered and washed with deionized water and then dried at 60°C for 48 h.

### Chemical Extraction of Chitin

Shrimp shells were treated with 1.5 mol/L HCl at a ratio of 1:10 (w/v) for 6 h at 25°C (No et al., 2003). After removing minerals, samples were washed in pure water until neutral. After the excess water was removed, they were put into the reflux device, and 20 ml of 10% NaOH was added to reflux (90°C) for 2–3 h. After the treatment, the samples were washed in pure water and then dried at 60°C for 48 h.

### Measurement of Parameters

The ash content was determined at 550°C (Healy et al., 2003). The total nitrogen content of the shrimp shells was determined using the Kjeldahl method (Sjaifullah and Santoso, 2016). The corrected protein content was calculated by subtracting the chitin nitrogen from the total nitrogen content and multiplying it by 6.25.

DP% was calculated by Eq. 1, as follows (Nasri et al., 2011):


(1) $$DP\%=\frac{\left[\left(P_{O}\times O\right)-\left(P_{R}\times R\right)\right]}{P_{O}\times O}\times 100$$

Where *P*_*O*_ and *P*_*R*_ are the protein concentrations (%) before and after fermentation; while *O* and *R* represent the mass (g) of the original sample and fermented residues on a dry weight basis, respectively.

DM% was evaluated by Eq. 2, as follows:


(2) $$DM\%=\frac{\left[\left(M_{O}\times O\right)-\left(M_{R}\times R\right)\right]}{M_{O}\times O}\times 100$$

Where *M*_*O*_ and *M*_*R*_ are ash content (%) before and after fermentation; *O* and *R* represent the mass (g) of the original sample and fermented residue on a dry weight basis, respectively (Hamdi et al., 2017a,b).

After fermentation, the samples were treated with 1.5 mol/L HCl for 5 h at 25°C and 10% NaOH under reflux conditions (90°C) for 2 h to remove protein and mineral completely. The solid residues were dried at 60°C for 48 h. Residual protein and mineral content were determined by the method of section “Measurement of parameters.” Chitin recovery and yield from the samples were determined by Eqs 3 (Doan et al., 2019) and 4 (Zhang et al., 2017), respectively, as follows:


(3) $$\mathrm{Chitin}\mathrm{recovery}\%=\frac{\mathrm{Dry}\mathrm{weight}\mathrm{of}\mathrm{chitin}\mathrm{in}\mathrm{residue}}{\mathrm{Dry}\mathrm{weight}\mathrm{of}\mathrm{sample}}\times 100$$

(4) $$\text{Chitin yield\% =}\frac{\text{Chitin weight in fermented products }\times \text{ weight of fermented products}}{\text{weight of shrimp shell}}\times \text{100}$$

### Scanning Electron Microscopy Analysis

The electron microscopic images of shrimp shells were taken by JEOL JSM-6700F scanning electron microscopy (SEM), and *L. acidophilus* fermentation was compared to display the effects of successive two-step fermentation on chitin in shrimp shell (Liu et al., 2014).

### Fourier Transform Infrared Analysis

The samples were measured using a Bruker VERTEX70 Fourier transform infrared spectrophotometer (FT-IR). Spectral scanning was performed in the wavelength region between 4000 and 400 cm^–1^ at a resolution of 4 cm^–1^ with a scan speed of 2 mm/s.

The absorption bands at 1655 and 3450 cm^–1^ were used to calculate the degree of deacetylation (DD%) according to Eq. 5 (Ben Seghir and Benhamza, 2017), as follows:


(5) $$DD\%=100-\left[\left(A_{1655}/A_{3450}\right)\times 115\right]$$

where *A*_1655_ and *A*_3450_ are the absorbances of samples at wavenumbers of 1655 and 3450 cm^–1^, respectively.

### X-Ray Diffraction Analysis

X-ray diffractograms on powder samples were obtained using a Rigaku D/max 2500/PC full-automatic X-ray diffractometer (XRD) where working conditions were 40 kV and 40 mA with Cu K_α1_ radiation at λ = 1.54 Å between 2θ angles of 5° and 70°. The scanning rate was 4.8° min^–1^ (Nasri et al., 2011).

The crystallinity index (I_*CR*_) was calculated by Eq. 6, as follows:


(6) $$I_{CR}=\left[\left(I_{110}-I_{am}\right)/I_{110}\right]\times 100$$

where *I*_*am*_ is the intensity of amorphous diffraction at 16°, and *I*_110_ is the maximum intensity at 20°.

### Statistical Analysis

The results are expressed as mean ± SD. Correlation and regression analysis were performed using the Origin 9.0 program. Correlation analysis by the phylogenetic tree was carried out using the SeqMan program.

## Results and Discussion

### Isolation and Identification of Bacterial Strains

**FIGURE 3:**
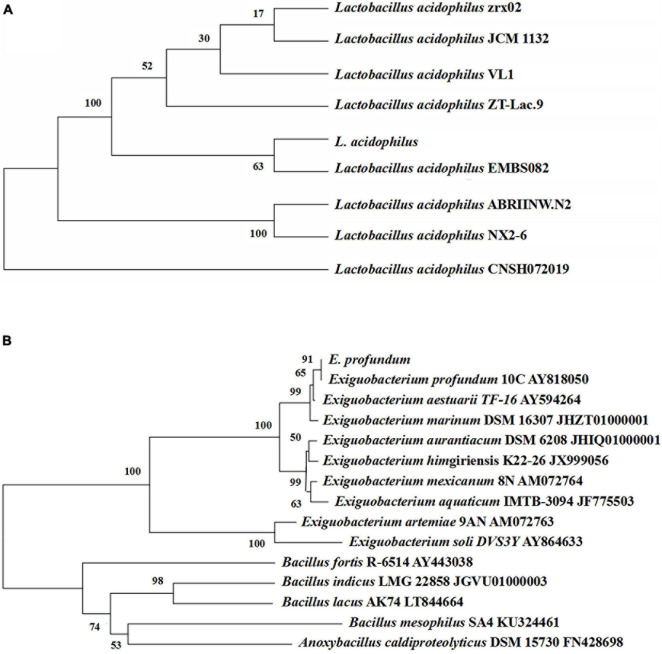
Phylogenetic tree based on the 16S rDNA gene sequences of *L. acidophilus* **(A)** and *E. profundum* **(B)**.

Thirteen strains of lactic acid-producing bacteria were isolated from traditional fermented shrimp paste, and one of the strains with high lactic acid yield was identified as *L. acidophilus* by 16S rDNA sequence analysis (Figure 3A). Ten strains with high protease activity were isolated from the traditional fermented shrimp paste, and one of these was identified as *E. profundum* using 16S rDNA sequence analysis (Figure 3B). The strain was found to belong to *Exiguobacterium* sp. by constructing a neighbor-joining phylogenetic tree. *E. profundum* can produce a high level of protease that hydrolyzes proteins in food. The present study showed that *E. profundum* could be cultivated in a medium containing shrimp shells as a sole nitrogen source.

### Analysis of *L. acidophilus* Fermentation Process

**FIGURE 4:**
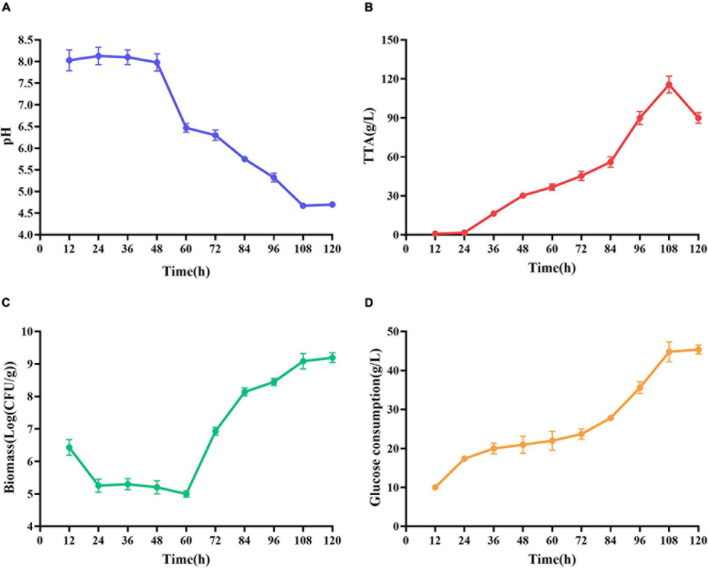
Changes in related parameters during fermentation of *L. acidophilus*. **(A)** pH; **(B)** total titratable acidity (TTA); **(C)** biomass; and **(D)** glucose consumption.

At the early stage of fermentation, pH = 8 was observed (Figure 4A). The pH = 8 was attributed to two reasons (Duan et al., 2012). Many amino acids and peptides produced buffering capacity by the hydrolysis of protease from shrimp shells and *L. acidophilus*. Carbonate in shrimp shells consume some lactic acids. The pH was decreased drastically within 48 h, and the amounts of total TTA and glucose consumption were gradually increased (Figures 4B,C). TTA may be closely related to lactic acid (Castro et al., 2018). Therefore, it could be reasonably assumed that TTA was directly derived from the lactic acid. After 108 h of fermentation, the TTA reached its highest point, and the increase in TTA reduced the pH of fermented liquor from 8.0 to less than 4.6. After 60 h of fermentation, the number of *Lactobacillus acidophilus* increased rapidly (Figure 4C) due to the logarithmic growth phase. It indicated that *L. acidophilus* was well adapted to growth in shrimp shell medium. After 108 h, the number of *L. acidophilus* was stable, and TTA and glucose consumption reached the highest level at this time (Figures 4B,D), indicating that *L. acidophilus* was inhibited in the acidic environment and gradually entered the autolysis stage. When microorganisms grew using available carbohydrate sources, lactic acid was released. The abundant lactic acid, which was responsible for DM, could dissolve CaCO_3_ to obtain water-soluble calcium lactate. Changes in glucose concentration were consistent with changes in *L. acidophilus* population, TTA, and lactic acid (Duan et al., 2012).

### Analysis of *E. profundum* Fermentation Process

**FIGURE 5:**
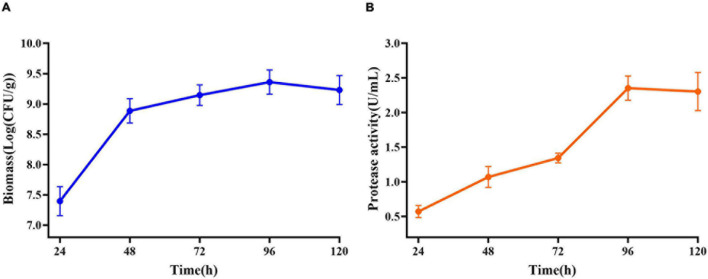
Changes in related parameters during fermentation of *E. profundum*. **(A)** Biomass; **(B)** protease activity.

Maximum cell growth reached after 96 h of fermentation (2.29 × 10^9^ CFU/mL) (Figure 5A). Maximum proteolytic activity was 4.65 U/ml, after 96 h, and then the activity decreased to 4.30 U/ml (Figure 5B) at the end of the process indicating that protease produced by *E. profundum* could hydrolyze proteins in shrimp shells and obtain carbon and nitrogen sources from the hydrolyzate. Therefore, shrimp shells can replace nutrients, which significantly reduces the cost of culture (Sedaghat et al., 2017).

### Successive Two-Step Fermentation With *L. acidophilus* and *E. profundum*

**FIGURE 6:**
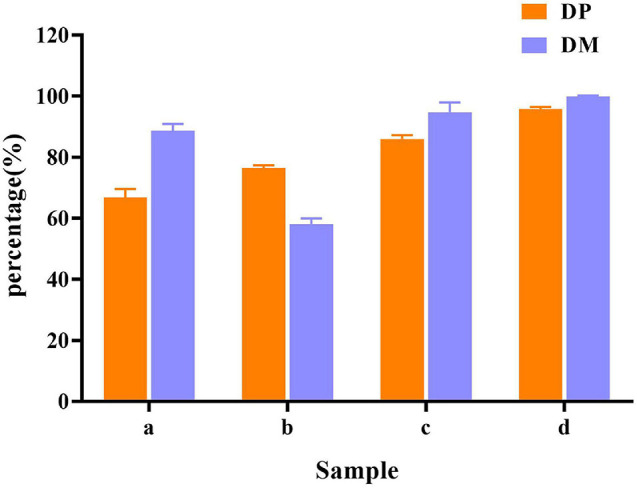
Demineralization (DM) and deproteination (DP) efficiency in different fermentation methods. (a) *L. acidophilus* fermentation; (b) *E. profundum* fermentation; (c) successive two-step fermentation; and (d) chemical extraction method.

In raw shrimp shells, protein (26.0 ± 0.53%) and ash (47.4 ± 2.2%) content were preferably high (Table 1), which are consistent with the previous studies (Ghorbel-Bellaaj et al., 2011; Hamdi et al., 2017a,b). The existence of proteins and ash with main components of minerals make shrimp shell hard, which blocks the release of chitin from shrimp shells. Decomposition or removal of proteins and minerals becomes a key procedure in the process of chitin extraction. Our results showed that the removing rate of minerals by *L. acidophilus* fermentation was 88.7 ± 2.2%. The degree of DM was higher than that reported by Rao and Stevens (2005) and was close to that reported by Flores-Albino et al. (2012) The degree of DP (66.8 ± 2.8%) was not satisfactory. *E. profundum* removed 76.4 ± 0.92% of protein, but the DM capacity (57.9 ± 2.0%) was weak. Successive two-step fermentation could remove 85.9 ± 1.2% of protein and 95 ± 3% of minerals (Figure 6). Chitin recovery and yield from samples were 47.82 and 16.32%, respectively (Table 2). The ash and protein content were reduced to 2.52 ± 0.25 and 4.65 ± 0.58%, respectively (Table 1). Low protein and mineral content strongly indicated the good quality of extracted chitins (Castro et al., 2018).

**TABLE 1:** Protein and ash content of the fermented residue.

**Sample**	**Protein (%)**	**Ash (%)**
Raw material	26.0 ± 0.53	47.4 ± 2.2
Residue of successive two-step fermentation	4.65 ± 0.58	2.52 ± 0.25

**TABLE 2:** Chitin recovery and yield of the fermented residue.

**Sample**	**Chitin recovery**	**Chitin yield**
Residue of successive two-step fermentation	47.82%	16.32%

Jung et al. (2006) extracted chitin from red crab (*Gecarcoidea natalis*) shell wastes by co-fermentation with *Lactobacillus paracasei* KCTC-3074 and *Serratia marcescens* FS-3. After 168 h of fermentation, the yields of DM and DP were 94.3 and 68.9%, respectively. Zhang et al. (2012) extracted chitin by successive two-step fermentation using *S. marcescens* B742 and *L. plantarum* ATCC 8014. After 6 days of fermentation, DP and DM reached 94.5 and 93.0%, respectively. Although the DP efficiency was slightly lower than the values reported in the same study, both *L. acidophilus* and *E. profundum* are safe organisms. *S. marcescens* acts as a conditional pathogen, but does not meet the requirements of the food industry. However, harmful acid caused depolymerization of the product. The inherent properties of chitin were changed, resulting in a decrease in its molecular weight and degree of acetylation. The intrinsic properties of purified chitin were also affected (Shamshina et al., 2016). The successive two-step fermentation helps to avoid many drawbacks of chemical treatment, which is a simple and environment-friendly alternative to chemical methods employed in the chitin extraction (Hajji et al., 2015).

### SEM Analysis

**FIGURE 7:**
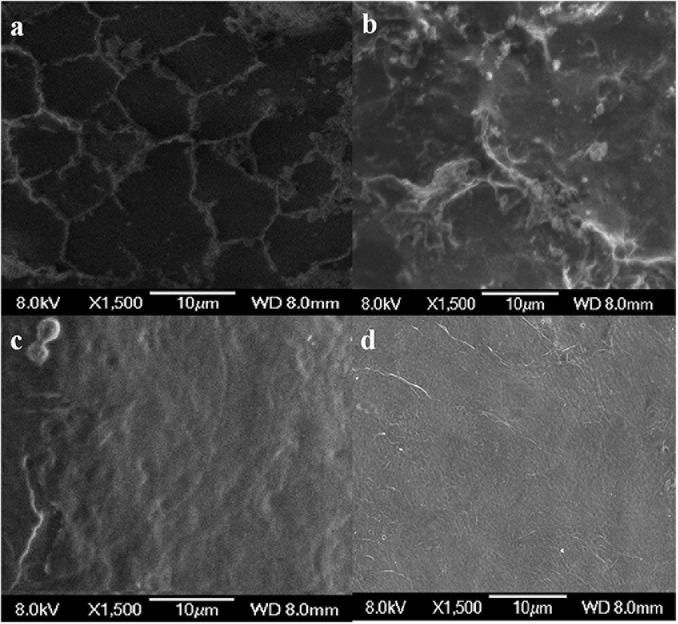
Scanning electron microscopy (SEM) micrographs of chitin obtained from shrimp shells **(a)**, *L. acidophilus* **(b)**, successive two-step fermented sample **(c)**, and chemical extraction method **(d)**.

The results of SEM showed that the surface of the shrimp shell was rough (Figure 7a). Many inorganic components were present and strongly embedded in chitin gaps and flexible protein macromolecules. *L. acidophilus* fermented sample (Figure 7b) surface was rough because of the presence of residual protein. The successive two-step fermented sample (Figure 7c) had a smooth surface, which has become uniform and porous with a lamellar organized structure (Flores-Albino et al., 2012). It was consistent with the description of Knidri et al. (2016), who found that the chitin structure showed several fine united leaves, with a highly porous, a lamellar organized and dense structure. It was similar to the chitin extracted by the chemical extraction (Figure 7d).

### FT-IR Analysis

**FIGURE 8:**
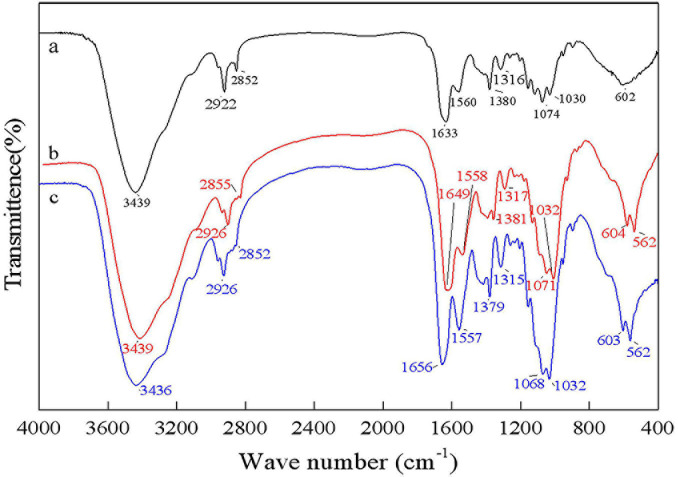
Fourier transform infrared spectrophotometer (FT-IR) spectrum for chitin obtained by chemical extraction method (a), *L. acidophilus* fermentation (b), and successive two-step fermentation (c).

As shown in Figure 8, the bands at 1656 cm^–1^ and 1380 cm^–1^ were C=O and C–H deformation bands, which are related to the acetamide group of chitin (Sedaghat et al., 2017). The band at 1320 cm^–1^ was an aliphatic C–H flexural vibration. The bands between 1250 and 800 cm^–1^ were related to the pyranoside ring (Rao and Stevens, 2005; Hajji et al., 2015). The DDs of samples after chemical extraction, *L. acidophilus* fermentation and successive two-step fermentation were 43.36, 8.11, and 3.67%, respectively. The density of peak area is positively correlated with the degree of acetylation (Zhang et al., 2017). These results were consistent with the calculated DD results. The high degree of deacetylation of chitin reflects the severity of its degradation (Younes et al., 2016). The low deacetylation degree of successive two-step fermented samples indicated that the deacetylation process of the chemical extraction of chitin was declined. Although pure chitin can be produced by chemical methods, the product obtained from this process can be a suitable product (Beaney et al., 2005). Biotechnological processes might be a viable option to overcome environmental and safety issues associated with chemical processes.

### XRD Analysis

**FIGURE 9:**
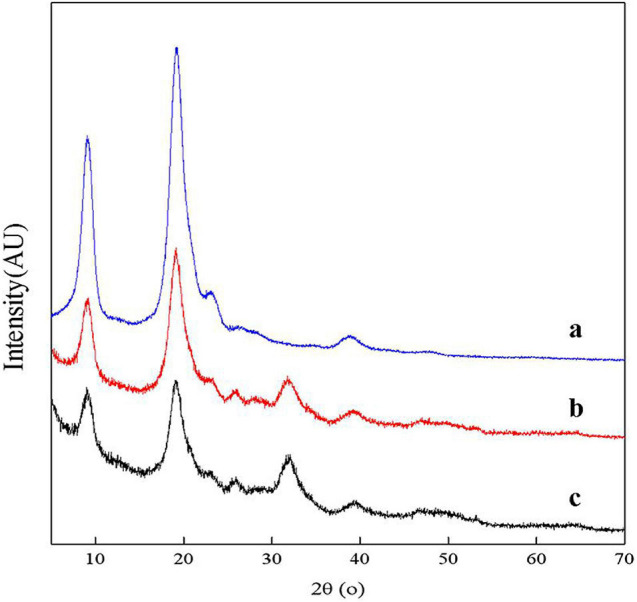
X-ray diffractometer (XRD) spectrum for chitin obtained by chemical extraction method (a), successive two-step fermentation (b), and *L. acidophilus* fermentation (c).

The XRD spectra of samples showed two main diffraction peaks at 9.3° and 19.2° and 3 weaks diffraction peaks at 12.9°, 23.4°, and 26.4° which are the characteristic peaks of the crystal lattice type of α-chitin as shown in Figure 9 (Jung et al., 2006). The diffraction peaks of CaCO_3_ at approximately 2θ = 29.55° were not shown, indicating that chitin extracted by fermentation contained no minerals. The *I*_*CR*_ values of chitin extracted by successive two-step fermentation, chemical method, and *L. acidophilus* fermentation were 54.37, 75.12, and 38.85%, respectively. The relatively low *I*_*CR*_ could be attributed to the breaking of intramolecular and intermolecular hydrogen bonds and the formation of amorphous chitin (Zhao et al., 2019), providing more possibilities for the subsequent chemical modification of chitin. In addition, fermentation broths rich in protein hydrolyzates (amino acids and polypeptides) should also be considered for further recycling (Todd, 1949).

## Conclusion

After successive two-step fermentation, 85.94 ± 1.22% of protein and 94.68 ± 3.25% of minerals were removed, with a chitin recovery of 47.82%. The low levels of residual minerals (2.52 ± 0.25%) and protein (4.65 ± 0.58%) indicated the superior quality of the extracted chitin. The chitin from successive two-step fermentation contained a low deacetylation degree, avoiding the chitin deacetylation process during the chemical extraction. Its low crystallinity value would provide the possibility for more efficient chemical modification in the subsequent processing steps. The method significantly reduced the use of required chemicals and produced a large amount of protein-rich fermentation broth with high nutritional value, which has great potential to produce high-value proteins for consumption. This study provides a relatively simple and environmentally friendly alternative method for preparing chitin from shrimp shells.

## Data Availability Statement

The original contributions presented in the study are included in the article/supplementary material, further inquiries can be directed to the corresponding author/s.

## Author Contributions

RX: conceptualization. WX: data curation. JY: writing—original draft preparation. JX: writing—review and editing. ZS and LS: supervision. XY: project administration. All authors have read and agreed to the published version of the manuscript.

## Conflict of Interest

XY was employed by company Qingdao Keda Future Biotechnology Co., Ltd. The remaining authors declare that the research was conducted in the absence of any commercial or financial relationships that could be construed as a potential conflict of interest.

## Publisher’s Note

All claims expressed in this article are solely those of the authors and do not necessarily represent those of their affiliated organizations, or those of the publisher, the editors and the reviewers. Any product that may be evaluated in this article, or claim that may be made by its manufacturer, is not guaranteed or endorsed by the publisher.
